# Pilsicainide Toxicity-Induced Brugada-Like ST Segment Elevation and Increased Pacing Voltage Threshold

**DOI:** 10.7759/cureus.51576

**Published:** 2024-01-03

**Authors:** Kosuke Hirose, Yoh Arita, Nobuyuki Ogasawara

**Affiliations:** 1 Department of Cardiology, Japan Community Healthcare Organization Osaka Hospital, Osaka, JPN

**Keywords:** bradycardia, pacing threshold, brugada-like ecg, toxicity, pilsicainide

## Abstract

Pilsicainide is a class Ic antiarrhythmic agent that exhibits fully selective sodium channel blockade. In Japan, it is one of the most prescribed medicines for rhythm control in atrial fibrillation. Pilsicainide is mainly excreted by the kidney. Therefore, the plasma concentration of pilsicainide is likely to be increased in patients with renal insufficiency. In this case report, a 90-year-old woman presented with generalized fatigue and loss of appetite. Her ECG showed marked bradycardia and coved-type ST-segment elevation similar to that of the Brugada type 1 pattern. Owing to dehydration, her renal function indices worsened compared with those measured four months prior. The plasma pilsicainide concentration was elevated to 2.67 µg/mL (therapeutic range: 0.20-0.90 µg/mL), indicating pilsicainide toxicity. A transvenous temporary pacemaker was placed; however, the pacing voltage threshold was increased at several sites within the right ventricle. Pilsicainide administration was immediately discontinued. On day 2 of admission, ventricular backup pacing was no longer required, and there was an improvement in renal function and heart failure symptoms, such as pulmonary edema and cardiomegaly. The ECG changes improved alongside the renal function and as the plasma concentration of pilsicainide decreased. In conclusion, elevated plasma concentrations of pilsicainide can induce life-threatening arrhythmias and pacing failure. Therefore, clinicians should prescribe pilsicainide cautiously, particularly in older patients.

## Introduction

Pilsicainide is a class Ic antiarrhythmic drug developed in Japan [1]. It is useful in restoring sinus rhythm in patients with recent-onset atrial fibrillation [2]. Pilsicainide is also useful for unmasking the ECG manifestations of the Brugada pattern through sodium channel blockade [3]. Since pilsicainide is primarily excreted by the kidneys, its plasma concentration is likely to increase in patients with renal dysfunction [4]. Elevated plasma concentrations of pilsicainide may induce life-threatening arrhythmias, such as sinus pause and ventricular tachycardia [5,6]. Moreover, these elevated levels may cause the Brugada-like ECG manifestation of coved-type ST-segment elevation [6,7]. This case report describes an older woman who developed pilsicainide toxicity due to renal dysfunction. Her ECG showed coved-type ST-segment elevation accompanied by bradycardia, and her temporary pacing voltage threshold was elevated. These features improved as her renal function recovered.

## Case presentation

A 90-year-old woman presented to our hospital with generalized fatigue and loss of appetite. A few days before her visit, she developed diarrhea and decreased appetite. Her medical history included paroxysmal atrial fibrillation, premature ventricular contractions, hypertension, and chronic kidney disease. She was prescribed pilsicainide (150 mg daily), cibenzoline (100 mg daily), and carvedilol (5 mg daily) for paroxysmal atrial fibrillation, along with amlodipine (5 mg daily) for hypertension.

**Figure 1:**
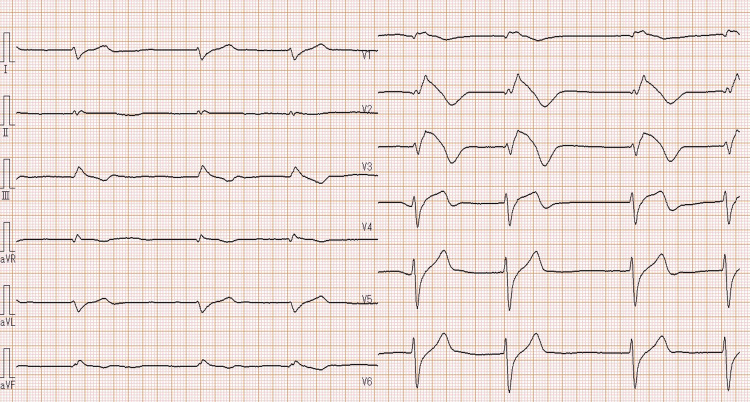
A 12-lead electrocardiogram showed slow atrial fibrillation (39 bpm), widened QRS (184 ms), long QTc (463 ms), and coved-type ST segment elevation in leads V1 to V3.

At presentation, the patient’s blood pressure was 97/63 mmHg. A 12-lead ECG showed slow atrial fibrillation (39 bpm), widened QRS (184 ms), long QTc (463 ms), and coved-type ST-segment elevation in leads V1 to V3 (Figure 1).

**Figure 2:**
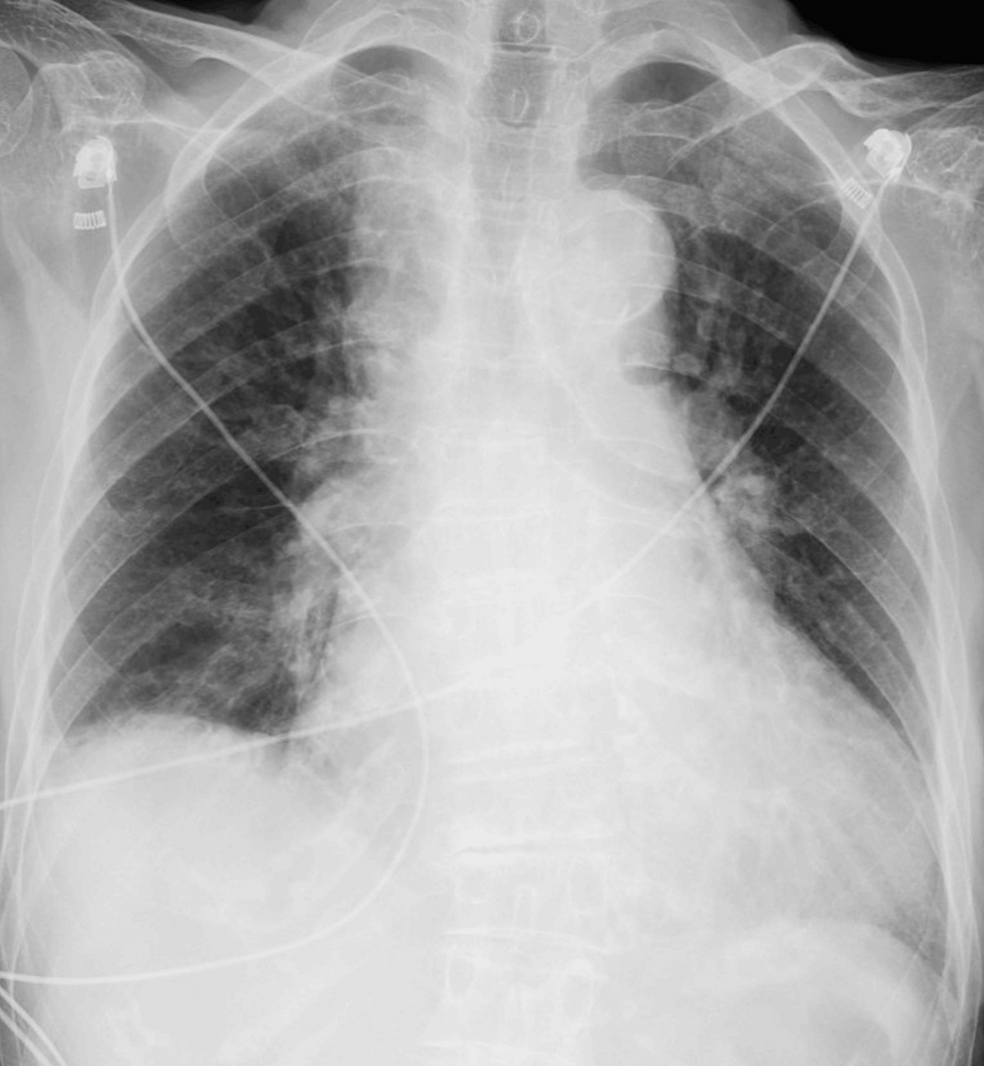
Chest radiograph revealed cardiomegaly and pulmonary edema.

A chest radiograph revealed cardiomegaly and pulmonary edema (Figure 2). Echocardiography showed dilatation of the inferior vena cava (21 mm) with poor respiratory variation, moderate tricuspid regurgitation, and pulmonary hypertension. Left ventricular wall motion was not impaired, and the left ventricular ejection fraction was 77%, as calculated using the Teichholz formula. Laboratory examinations were performed, revealing a blood urea nitrogen level of 47 mg/dL (normal range: 8-20 mg/dL) and a serum creatinine level of 1.61 mg/dL (normal range: 0.4-1.1 mg/dL). Worsening renal function was evident, as the serum creatinine level was 0.94 mg/dL four months prior to the patient's hospital visit. The serum potassium level was slightly elevated at 5.3 mEq/L (normal range: 3.5-5.0 mEq/L). The serum N-terminal pro-B-type natriuretic peptide (NT-proBNP) level had increased to 1,945 pg/mL (normal range: <125 pg/mL). The plasma pilsicainide concentration was elevated at 2.67 µg/mL (therapeutic range: 0.20-0.90 µg/mL), and the plasma cibenzoline concentration was 0.398 µg/mL (therapeutic range: 0.20-0.80 µg/mL). Therefore, we diagnosed the patient with pilsicainide toxicity secondary to worsening renal failure and heart failure, accompanied by bradycardia. 

A transvenous temporary pacemaker was inserted through the right internal jugular vein into the right ventricle. Despite repeated attempts to position the lead at several sites on the right ventricular wall, a pacing voltage threshold of less than 2.0 V could not be achieved. Therefore, the pacing output voltage and pacing rate were adjusted to 5.0 V and 60 bpm, respectively.

**Figure 3:**
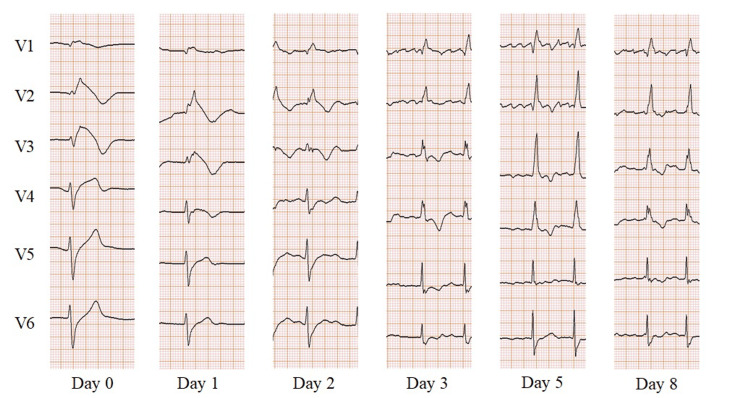
Electrocardiogram changes throughout the course of treatment.

Pilsicainide administration was immediately discontinued, and 20 mg of furosemide was administered intravenously. Twelve hours after admission, the ECG showed atrial fibrillation with a rate of 87 bpm. As a result, her temporary pacemaker was no longer necessary and was removed on day 2 of hospitalization. By day 3 of hospitalization, her serum creatinine level improved to 0.83 mg/dL, which was identical to the level observed before admission. Oral administration of azosemide (30 mg) was initiated to manage residual volume overload, and the dose was reduced to 15 mg on day 9 of hospitalization. On day 8, the plasma pilsicainide concentration was lower than the detection limit, and no ST-segment elevation was detected on her ECG. The changes in her ECG throughout the treatment course are presented in Figure 3. The patient responded well to treatment for pilsicainide toxicity and heart failure with preserved ejection fraction; however, she developed pseudogout on day 9 of hospitalization. Although she required pain medications and rehabilitation, she was discharged on day 18 of hospitalization.

## Discussion

Pilsicainide, classified as a class Ic antiarrhythmic agent according to the Vaughan-Williams classification, exhibits fully selective sodium channel blockade. It is effective in restoring sinus rhythm in patients with recent-onset atrial fibrillation [2]. In Japan, pilsicainide is one of the most commonly used medications for rhythm control in atrial fibrillation. Because it strongly blocks sodium channels, various ECG changes can be triggered by its administration. An increased plasma level of pilsicainide can cause lethal arrhythmias, such as sinus pause and ventricular tachycardia [5,6]. Although the therapeutic blood concentration of pilsicainide is 0.2-0.9 µg/mL, the toxic level is variable. In a previous report, patients with toxicity had pilsicainide concentrations ranging from 1.89 µg/mL to 14.9 µg/mL [8]. Since pilsicainide is primarily excreted by the kidneys, its duration of action is prolonged in patients with renal dysfunction [4]. Drug-induced arrhythmias caused by pilsicainide are strongly associated with renal dysfunction [9]. Although our patient was elderly and had chronic kidney disease, she was prescribed a typical dose of pilsicainide. Her renal function deteriorated due to dehydration caused by diarrhea and loss of appetite, leading to an increase in her blood concentration of pilsicainide above the therapeutic range. Consequently, she developed bradycardia-related heart failure. In a previous report, it was noted that the pacing voltage threshold in patients with pacemakers increased as the blood concentration of pilsicainide rose [10]. In our case, the pacing voltage threshold of the right ventricular temporary pacemaker was elevated at several sites, leading us to suspect pilsicainide toxicity, accompanied by bradycardia and heart failure. One day after admission, ventricular backup pacing was no longer necessary, and there was an improvement in both renal function and heart failure.

Pilsicainide is used as a pharmacological test to diagnose Brugada syndrome by unmasking coved-type ST-segment elevation [3]. Pilsicainide toxicity may induce ST-segment elevation similar to that seen in Brugada syndrome [7]. In three reported cases, the pilsicainide concentrations were 2.85, 2.50, and 4.18 µg/mL. In our case, the pilsicainide concentration was similar to or lower than those in the reported cases. However, the patient's ECG showed marked bradycardia and Brugada-like ST-segment elevation. Cibenzoline, another sodium channel blocker, might have also contributed to and enhanced the Brugada-like ECG changes, even though the cibenzoline concentration was within the therapeutic range [3]. The ECG changes improved alongside the improvement in renal function and the decrease in blood pilsicainide concentration.

## Conclusions

The plasma pilsicainide concentration can rise to a toxic level in older patients who have decreased renal functional reserve during episodes of dehydration due to loss of appetite and diarrhea. Therefore, clinicians should prescribe this drug cautiously in older patients.
